# Antibacterial Effect of Ozone on Cariogenic Bacteria and Its Potential Prejudicial Effect on Dentin Bond Strength—An In Vitro Study

**DOI:** 10.3390/pharmaceutics16050614

**Published:** 2024-05-02

**Authors:** Marta Santos, Flávia Leandro, Helena Barroso, António H. S. Delgado, Luís Proença, Mário Polido, Joana Vasconcelos e Cruz

**Affiliations:** 1 Egas Moniz School of Health and Science, Monte da Caparica, 2829-511 Almada, Portugal; marta.filipa00@hotmail.com (M.S.); flaviasofialeandro@gmail.com (F.L.); mhbarroso@egasmoniz.edu.pt (H.B.); aldelgado@egasmoniz.edu.pt (A.H.S.D.); lproenca@egasmoniz.edu.pt (L.P.); mpolido@egasmoniz.edu.pt (M.P.); 2 Egas Moniz Center for Interdisciplinary Research (CiiEM), Monte da Caparica, 2829-511 Almada, Portugal

**Keywords:** ozone, dental caries, anti-bacterial agents, *Streptococcus mutans*, *Streptococcus sobrinus*, *Lactobacillus casei*, *Actinomyces*, bond strength, dentin

## Abstract

Ozone is increasingly utilized in dental caries treatment due to its antibacterial properties. In a context of limited studies and no consensus on protocols, this research aims to assess ozone’s antibacterial efficacy on cariogenic bacteria and its potential adverse impact on dentin bond strength. *Streptococcus mutans*, *Streptococcus sobrinus*, *Lactobacillus casei*, and *Actinomyces naeslundii* suspensions were exposed to 40 μg/mL of ozone gas and 60 μg/mL of ozonated water (80 s) via a medical ozone generator. Negative and positive control groups (chlorhexidine 2%) were included, and UFC/mL counts were recorded. To examine microtensile bond strength (µTBS), 20 human molars were divided into four groups, and class I cavities were created. After ozone application, samples were restored using an etch-and-rinse and resin composite, then sectioned for testing. The SPSS v. 28 program was used with a significance level of 5%. The µTBS results were evaluated using one-way ANOVA, Tukey HSD, and Games-Howell. Bacterial counts reduced from 10^6^ to 10^1^, but dentin µTBS was significantly impacted by ozone (ANOVA, p < 0.001). Despite ozone’s attractive antibacterial activity, this study emphasizes its detrimental effect on dentin adhesion, cautioning against its use before restorative treatments.

## 1. Introduction

A paradigm shift is taking place in dentistry, mainly in the way that dentists approach their practice. Today, there is greater concern to minimize the need for restorative practices, as well as to carry out more conservative and biocompatible treatments, focused on preserving the remaining dental tissue [1,2,3].

Dental caries, one of the most prevalent diseases worldwide, is also one of the primary reasons for dental restoration failure, resulting from bacterial infiltration around the restoration margins. Various strategies have been investigated to address this issue and increase the longevity of restorations. This approach often encompasses the use of antibacterial topical agents, aimed at reducing the levels of microorganisms in dental plaque or the remaining tooth after the removal of decayed tissue, potentially reducing the risk of secondary caries, post-operative sensitivity, and pulp inflammation [4,5,6,7].

Additionally, emerging technologies such as nanomaterials and bioactive compounds show potential for enhancing the durability and performance of restorations. There is a growing emphasis on developing biologically compatible materials with fewer chemical components in dental restoration research, reflecting a more holistic approach to restorative dentistry [8]. In this context, ozone therapy has increasingly garnered attention from healthcare professionals.

Ozone is an unstable, colorless gas composed of three oxygen atoms (O_3_) which exhibits disinfectant properties against bacteria, fungi, and viruses [5,6]. It causes the oxidation of phospholipids and lipoproteins in the bacterial cell envelope, destroying the cell wall and rupturing the cytoplasmic membrane, allowing the ozone itself to infiltrate the microorganisms. Consequently, it oxidizes glycoproteins and glycolipids, thus blocking enzyme function, which leads to a loss of organelle function [7,8,9,10]. For this reason, ozone could be promising in conservative dentistry since it could potentially play a role in the prevention of dental caries and as a cavity disinfectant [7,9,10,11,12].

Ozone therapy in dentistry has been increasingly applied in diverse areas such as periodontology, endodontics, dentistry, and surgery due to its obvious disinfecting power. This compound can be used to treat cavities, gingivitis and periodontitis, oral lichen planus, osteonecrosis, endodontic treatment, halitosis, and for pain control [13,14].

However, the protocol for ozone application (forms, concentrations and time) with efficacy against cariogenic bacteria is still not clear in the literature. The studies that were found show a significant disparity in the results published, the methodology studied, and the equipment used [6,15,16,17,18]. Besides, although there are some in vitro studies of the antimicrobial capacity of ozone and how effective it is, in most of them, ozone is applied to biofilms and/or human teeth, having a strong mechanical action that could interfere with the results [17,18]. As a consequence, there is an extra demand to test the action of ozone on microorganisms in a controlled environment without mechanical action.

Due to its properties, the application of ozone prior to dental restorations presents an intriguing and promising procedure, as it has the potential to preserve more dental structure while enhancing the longevity of the restoration through its antibacterial action. However, due to the instability of ozone, it decomposes into oxygen free radicals that react with the polymerization monomers, due to the high reactivity of oxygen. For this reason, ozone has the ability to interfere with the polymerization reaction in restorative materials, leading to a decrease in bond strength [17]. Unfortunately, there are still few published studies on the interference of ozone in the adhesive restorative procedure, and also on the resulting bond strength. The introduction of ozone generators that use medical oxygen to form ozone allows this compound to be applied in higher concentrations compared to generators that use ambient air. For this reason, it is urgently necessary to carry out a study to clarify whether the application of ozone, under these clinical conditions, has negative or positive effects on bond strength.

Therefore, the objectives of the following study are to assess in vitro the antibacterial effect of different ozone concentrations and forms (gas, water, gas+water) on cariogenic bacteria without any mechanical action and evaluate in vitro the microtensile bond strength in dentin when different forms of ozone are applied immediately after a tooth restoration.

## 2. Materials and Methods

### 2.1. Antibacterial Effect on Cariogenic Bacteria

A total of 8 independent tests were performed, each in triplicate. For two independent tests, in addition to the three replicate tests, plating for counting CFU/mL was also carried out in triplicate. Each independent test, for each microorganism, was carried out in triplicate and included the experimental groups and, simultaneously, a negative control group, in which the bacteria were not subjected to any treatment, and a positive control group, in which 2% chlorhexidine was applied.

The microorganisms *Streptococcus mutans*, *Streptococcus sobrinus*, *Lactobacillus casei* and *Actinomyces naeslundii* were used in these experiments due to their known role in the development of caries [19]. They were maintained as recommended by the curator of the appropriate culture collection.

Suspensions of *Streptococcus mutans* ATCC 35668, *Streptococcus sobrinus* DSM 20742, *Lactobacillus casei* ATCC 393, and *Actinomyces naeslundii* DSM 43013 were exposed to 40 μg/mL of pure gaseous ozone and 60 μg/mL of ozonated water during 80 s through a medical ozone generator machine “Ozonette Dent” (Sedecal, Madrid, Spain). The ozone concentration and application time were selected according to the guidelines of the Madrid Declaration on Ozone Therapy and the Sedecal company, which markets the generator, and allows a range of concentrations from 1 to 80 μg/mL [11].

In order to ensure the standardization of the suspensions, the McFarland DEN-1 densitometer (Grant Instruments, Cambridgeshire, United Kingdom) was used, in which an approximate microbial content of 10^8^ CFU/mL (McFarland scale 1) was verified.

**Figure 1:**
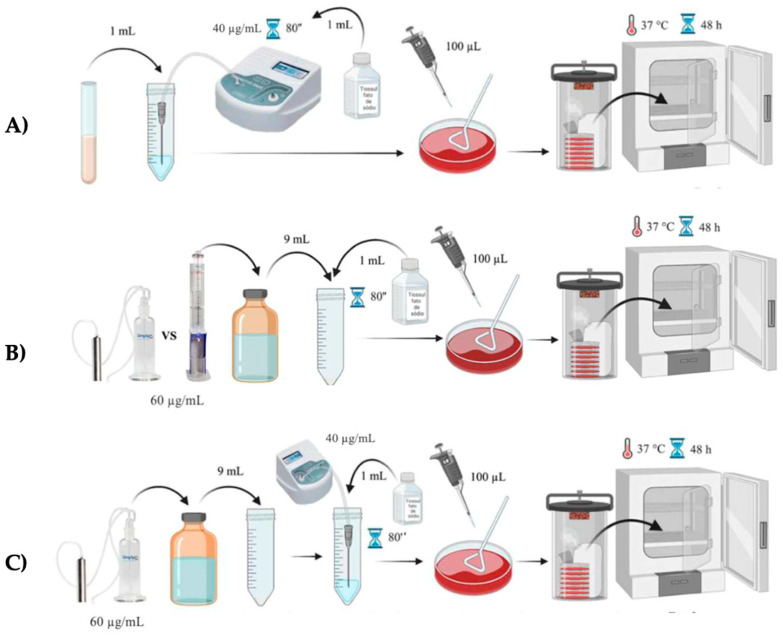
Representative scheme of the experimental ozone application protocol. (**A**) Ozone gas group; (**B**) ozone water group; (**C**) ozone gas + ozone water group.

As illustrated in Figure 1, 1 mL of the bacterial suspension was diluted in 9 mL of sterile Milli-Q ultrapure water (Merck-Millipore, Darmstadt, Germany). The gaseous ozone was applied directly to the diluted suspension using an extender and a Sterican irrigation needle (B. Braun, Melsungen, Germany) connected to the Ozonette Dent generator (Sedecal, Madrid, Spain) in continuous mode at a concentration of 40 μg/mL, with a flow rate of 30 L/h. After 80 s, 1 mL of 10% sodium thiosulfate (VWR, Radnor, USA) was added to inactivate the ozone. Afterwards, 100 μL of the experimental mixture was inoculated on specific culture medium (blood agar for *Streptococcus*, chocolate agar (CHOC) for *Actinomyces*, and MRS (De Man–Rogosa–Sharpe) agar for *Lactobacillus*) and placed in the incubator at 37 °C for 48 h (microaerophilic conditions for *Lactobacillus* and anaerobic conditions for the others).

The ozonated water was made in two different ozone systems: an ozone water bubbler system (SimplyO_3_, Grand Ledge, USA); and a microbubble water ozonation column (Philozon, Nova Esperança, Brazil) connected to the Ozonette Dent generator (Sedecal, Madrid, Spain) in continuous mode at a concentration of 60 μg/mL, with a flow of 30 L/h for 5 min (Figure 1). Sterile Milli-Q ultrapure water (Merck-Millipore, Darmstadt, Germany), at a temperature of 4 °C, was used for the ozonation and it was stored in an amber glass bottle and used immediately to guarantee a maximum and stable concentration of ozone in the water. A quantity of 500 mL of sterile Milli-Q ultrapure water (Merck-Millipore, Darmstadt, Germany) was ozonated in the ozone water bubbler system (SimplyO_3_, Grand Ledge, USA) and 1 L was ozonated in the microbubble water ozonation column (Philozon, Nova Esperança, Brazil). In these experimental groups, 9 mL of the ozonized water was mixed directly into 1 mL of the bacterial suspension, and after 80 s 1 mL of 10% sodium thiosulfate (VWR, Radnor, USA) was added to inactivate the ozone. Afterwards, 100 μL of the experimental suspension was inoculated on specific culture medium and placed in the incubator at 37 °C for 48 h.

The simultaneous application of gaseous ozone and ozonized water, with the methods detailed above, was also tested in order to investigate their synergistic action (Figure 1). The results were determined by counting the number of CFU (colony forming unit) on the inoculated media. In cases of no bacterial growth, the reference value used was <10 CFU/mL.

### 2.2. Microtensile Bond Strength Test on Dentin after Ozone Application

After approval by the ethics committee of the Egas Moniz School of Health and Science (no. 1137), twenty sound human molars, free of carious lesions, structural defects, or restorative treatments, extracted within the last 6 months, were collected and stored in 0.5% chloramine T trihydrate, followed by distilled water until the start of the study. In line with Armstrong et al. (2017) [20], to calculate the sample number, five teeth were selected for each of the four experimental groups in order to achieve statistical relevance.

To separate the coronary portion from the root, the samples were cut perpendicular to the long axis of the tooth, below the amelocementary junction, using a microtome (Accutom-50, Struers A/S, Ballerup, Denmark). Afterwards, the dental pulp was removed with a dentine spoon and the pulp chamber was filled with cyanoacrylate glue. Class I cavities were then made using a special equipment with a parallelometer coupled to a high-speed handpiece, allowing standardization [21]. The cavity was made with a straight-ended cylindrical burr and abundant water irrigation, to obtain a final cavity configuration size of 2.5 mm depth and a length and width of 4 mm × 5 mm [22].

Samples were then randomly divided into 4 groups (*n* = 5): (1) CTR–Control (no ozone application); (2) O_3__GAS–Gaseous ozone with a concentration of 40 µg/mL for 80 s; (3) O_3__H_2_O − 100 mL of water ozonized with a concentration of 80 µg; (4) O_3__H_2_O + GAS − 100 mL of water ozonized with a concentration of 80 µg/mL, followed by application of gaseous ozone with a concentration of 40 µg/mL for 80 s.

**Figure 2:**
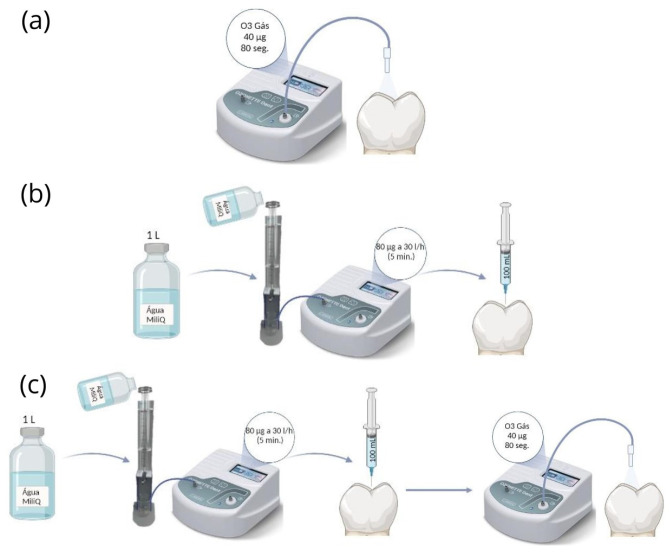
Representative step-by-step diagram of ozone application. (**a**) O_3__GAS group; (**b**) O_3__AG group; (**c**) O_3__AG + GAS group.

As illustrated in Figure 2, the ozone gas was formed using an Ozonette Dent generator (Sedecal, Madrid, Spain) coupled to a medical oxygen bottle, with a concentration of 40 µg/mL, and was applied inside the cavity for 80 s. To form the ozonated water, 1 L sterile Milli-Q ultrapure water (Merck-Millipore, Darmstadt, Germany), at a temperature of 4 °C, was placed in the column (Philozon, Nova Esperança, Brazil) and the generator was set to a concentration of 80 µg/mL at 30 L/H during 5 min. After ozonating the water, 100 mL was placed inside the cavity for 80 s. Finally, the O_3__H_2_O+GAS group allows the combination of the two forms of ozone to verify their synergistic effect. In this group, ozonized water was applied first, followed by gaseous ozone, since gaseous ozone is more dissolvable in aqueous environments.

For all samples, the Optibond^TM^ FL adhesive system (Kerr Corporation, Orange, CA, USA), was applied according to the manufacturer’s instructions. First, 37% orthophosphoric acid was applied to the enamel and dentin for 15 s, followed by a 15 s rinse and careful drying. Next, the primer was applied using circular movements for 15 s and then dried for 5 s. Finally, the bond was applied for 15 s, dried for 5 s, and light-cured for 10 s using the light-curing device Elipar^TM^ DeepCure-S (3M ESPE, Seefeld, Germany) with an intensity of 900 mW/cm^2^.

A 5.5 mm resin build-up was made with 2 mm increments of Filtek Z250 resin shade A2 (3M ESPE, Seefeld, Germany) in the cavity. The increments were light-cured for 20 s with the Elipar^TM^ DeepCure-S light-curing device (3M ESPE, Seefeld, Germany). The samples were then stored in distilled water for 24 h and placed in an incubator (Memmert INE 400, Schwabach, Germany) at 37 °C [20].

After 24 h, the samples were sectioned using a microtome (Accutom-50, Struers A/S, Ballerup, Denmark) to obtain sticks with a dimension of 1 ± 0.3 mm^2^. Enamel specimens were discarded. The size of the sticks was measured using a digital stud (Vogel Germany, Kevelaer, Germany) to calculate the cross-sectional area of the adhesive interface. Afterwards, the sticks were bonded with cyanoacrylate glue to stainless steel jigs to be tested for microtensile forces [20].

A universal testing machine (Shimadzu, Autograph AG-IS, Tokyo, Japan) at a speed of 0.5 mm/min and a load cell of 0.5 N was used. The failures obtained were classified as adhesive, cohesive, and mixed, and only adhesive fractures were considered for microtensile bond strength (µTBS) evaluation. Pre-test failures were recorded with a value of 0 MPa [20].

The SPSS v. 28 program was used to evaluate dentin µTBS, with a significance level of 5%. The groups were evaluated using one-way ANOVA, Tukey HSD, and Games-Howell.

## 3. Results

### 3.1. Antibacterial Effect on Cariogenic Bacteria

**Figure 3:**
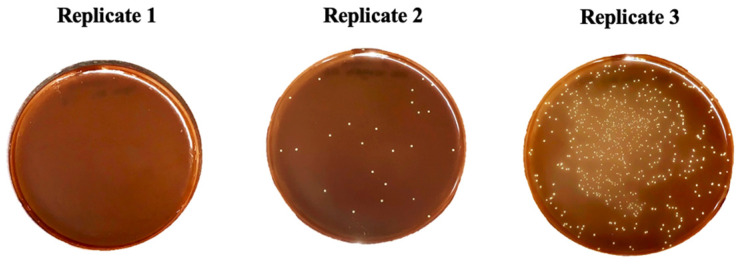
Example of heterogeneity in the number of CFU/mL between replicates of the S. mutans test in the O3_GAS80” group.

When counting the number of Colony Forming Units (CFU), it was found that 2% chlorhexidine eliminated 100% of all microorganisms, validating the study methodology. However, the ozone groups showed heterogeneous results between triplicate experiments (Table 1 and Figure 3).

Nevertheless, in comparison with the initial number of bacteria, there were always reductions, ranging from 10^6^ to 10^1^, in all the bacteria tested and for all methods of ozone application. Thus, the arithmetic average of the colony forming units (CFU/mL) is shown in Table 2.

### 3.2. Microtensile Bond Strength Test on Dentin after Ozone Application

**Figure 4:**
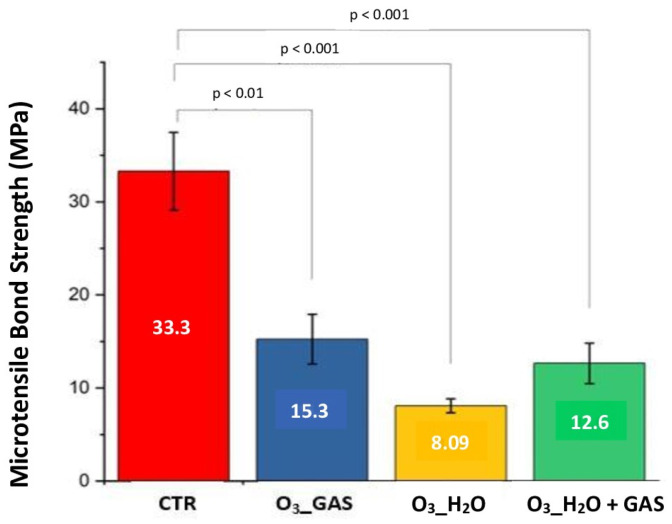
Bar chart comparing the four experimental groups in relation to the microtensile bond strength test (MPa). The CTR group average (33.3 MPa) is significantly higher compared to the O_3__GAS group (15.3), O_3__H_2_O (8.09 MPa), and O_3__H_2_O + GAS (12.6 MPa). Thus, there were significant differences between the CTR group and the other ozone groups. The error bars shown correspond to the standard errors of the means.

The results of the µTBS test are shown in Figure 4. The one-way ANOVA test confirmed statistically significant differences (Z = 16.255; *p* < 0.001), since varying the treatment had an impact on the microtensile bond strength. To identify differences, Games-Howell was used, which found differences between the control group and the O_3__GAS (*p* < 0.01), O_3__H_2_O (*p* < 0.001), and O_3__H_2_O + GAS (*p* < 0.001) groups. However, there were no significant differences between the ozone treatment groups, even in distinct forms.

The final counts of the different types of failure at the resin-dentin interface (adhesive, cohesive, or mixed failure), identified by percentages, are detailed in Table 3.

## 4. Discussion

Although this study confirmed the antibacterial efficacy of ozone in both gas and water forms against the studied cariogenic bacteria, the results revealed some variability due to ozone’s high reactivity and a significant negative influence on adhesive efficacy when applied before restoration. This serves as a cautionary note for healthcare professionals who routinely utilize this therapeutic tool.

The dynamic process of caries lesions consists of alternating periods of dental demineralization and remineralization. A balance between pathological and protective factors is paramount, so that the disease stabilizes or even reverses, in initial cases [19]. In this sense, some authors state that correct oral hygiene, controlling normal salivary function rate, reducing the intake of fermentable carbohydrates, taking prebiotics and probiotics, and applying sealants, remineralizing and antibacterial agents will cause the caries lesion to stabilize and promote remineralization [23,24,25,26].

As a result, ozone has been studied in dentistry due to its oxidizing and remineralizing properties and due to the fact that it is a simple, non-invasive therapy that can reduce the total appointment time, which is especially important for those who suffer from phobia, making it very interesting in pediatric dentistry [4].

This study showed that the application of ozone for 80 s has antibacterial activity against the cariogenic bacteria studied, both in the form of ozonated water and ozone gas. However, there were numerous oscillations between the triplicates within the groups. One of the explanatory hypotheses for these oscillations is the low solubility of gaseous ozone in water, resulting in rapid self-decomposition and a decrease in its action [26]. The dissolution of ozone in liquid media is significantly affected by pH, decreasing its dissolution at alkaline pH > 8, as is the case in our study, where the solution had a pH of 9.3. Another hypothesis is that ozone formed clusters and did not dissolve and disperse correctly in water, which is why not all bacteria are affected [27]. The third explanatory hypothesis is the formation of bacterial clusters due to the oxidative stress caused by ozone. If there are compact microbial agglomerates, the ozone would have no action inside them, resulting in a reduction of action and in variations between counts of the number of CFUs in different places in the same liquid medium and between tests. This hypothesis is corroborated by the occurrence of the phenomenon of bacterial self-aggregation caused by stress, which remains poorly understood, as described in Trunk et al. (2018) [28].

The available literature on the antibacterial action of ozone on cariogenic bacteria is quite limited and insufficient to allow an adequate comparison of the results, since in most in vitro studies the ozone is applied to biofilms and/or human teeth with a mechanical action, as well as using different concentrations and application times [16,17,18]. The use of different generators also makes it impossible to develop a standard protocol. Nevertheless, our results are consistent with those published by Dukić et al. (2013) [16], Johansson et al. (2009) [17], and Kapdan et al. (2013) [18], where ozone showed a reduction in cariogenic bacteria, such as *S. mutans*, *S. sobrinus*, *L. casei*, and *A. naeslundii*. On the contrary, Sancakli et al. (2018) [6] state that the application of gaseous ozone for 80 s is not sufficient to achieve an effective antibacterial effect on *S. mutans*, although it is important to note that the article does not reveal the concentration of ozone used, which may have an important bearing on the results obtained.

Studies also state that there is a dilemma in the use of ozone prior to restoration due to its ability to influence the bond strength of composite resins [29,30]. The instability of ozone causes it to dissociate into oxygen molecules, which are one of the factors responsible for inhibiting the polymerization of composite resins and compromising adhesive strength [30]. Thus, according to the results obtained, the application of ozone prior to definitive restoration negatively influences the microtensile bond strength of dentin, so the study hypothesis is rejected. This may be due to the dehydration of dentin caused by ozone gas and the presence of residual oxygen after the application of ozone, since oxygen reacts with the monomer chain, interfering with polymerization [15,29,30].

Similarly to this study, Can et al. (2022) [29], Dalkilic et al. (2012) [31], and Rodrigues et al. (2011) [15] reported that the application of ozone prior to the restorative procedure decreased the bond strength of dentin to microtensile strength due to the presence of residual oxygen. The following studies used a similar method, applying only the adhesive system to a control group and applying ozone before the final restoration. However, only one study [31] has used a generator that produces ozone from medical oxygen with an unknown concentration. Therefore, although the results are similar, there is an urgent need to carry out more studies using a generator with medical oxygen with defined concentrations that can be replicated clinically.

On the other hand, the subject remains highly controversial because there are other articles which conclude that the application of ozone does not interfere with dental adhesion.

Since there are still no studies with a standardized protocol, there is a certain disparity between the concentrations of ozone administered and the time of application. This can be seen in the studies by Garcia et al. (2012) [30] and Oznurhan et al. (2015) [32], as they used a lower concentration and application time, which explains the lack of interference in bonding efficacy.

Cadenaro et al. (2009) [33], Oznurhan et al. (2015) [32], and Kapdan & Öztaş (2013) [34] presented different results. One of the reasons is the fact that they used a different generator, such as the HealOzone generator (KaVo, Biberach/Riss, Germany), which forms ozone from ambient air, so the amount of ozone produced will be significantly lower than when using medical oxygen.

Another reason for the difference is the use of a different adhesive system, such as self-etch. The self-etch system demineralizes and dehydrates the dentin less than the etch-and-rinse system used in this study, and therefore there is less collapse of the collagen fibers that contributes to a better dentin bonding, as was found in Cadenaro et al. (2009) [33].

Although there are several publications about ozone and its efficacy in the dentistry field, there are still few published studies on the effect of ozone on cariogenic bacteria, and its effect on dental adhesion. For this reason, more in vitro studies are needed to test the antibacterial action of ozone on other bacterial strains and study the most effective and reproducible concentration, time, and form of application for most cariogenic bacteria. It is also essential to establish a standardized protocol for better clinical application without interfering with dental adhesion. As ozone is a gas, its high reactivity makes it difficult to control the actual concentration that comes into contact with bacteria, and we highlight this aspect as a possible limitation of this study.

In the future, it is crucial to carry out a larger number of studies using well-defined and reproducible protocols in order to test the antibacterial action of ozone with long-term monitoring on other bacterial strains, and to use a kit that can measure the final concentration of ozone in ozonated water so that there is a better understanding of the dissolution of ozone in liquid environments. It would also be pertinent to evaluate the adhesive strength of dentin after the application of ozone followed by an antioxidant or to evaluate the effect of ozone on adhesive strength one week after the application of ozone.

## 5. Conclusions

Given the interest of ozone in dentistry and the increased use of ozone in clinical practice, this study intended to be innovative and to test the isolated antibacterial action of ozone, without mechanical action. Despite the limitations of the study, it is possible to conclude that ozone has an antibacterial effect against certain bacterial strains, namely *S. mutans*, *S. sobrinus*, *L. casei*, and *A. naeslundii*. However, the results obtained in microtensile bond strength to dentin suggest that ozone, regardless of the form it takes (gas or water), interferes with microtensile bond strength.

Therefore, it is important to alert dentists to the effects of the compound on the dental surface and highlight the need to carry out more studies that develop protocols that can combine the antibacterial properties of ozone without compromising dentin bond strength. For this reason, it would be interesting to carry out new studies that could investigate the effect of ozone on adhesive resistance after the application of an antioxidant or evaluate it one week after the application of ozone. To verify the concentration of ozone present in the water, it would be essential to carry out studies using a kit that measures the concentration of ozone.

## Figures and Tables

**Table 1:** Example of heterogeneity in the number of CFU/mL between replicates of the S. mutans test.

	Replicate 1	Replicate 2	Replicate 3
**O_3__GAS80″**	<10	250	5150
**O_3__H2O80″ Simply**	90	<10	9140
**O_3__H_2_O80″ Philozon**	210	5520	920
**O_3__H_2_O+GAS80″ Simply**	<10	<10	400

**Table 2:** Arithmetic average of the colony forming units (CFU/mL).

	*S. mutans*	*S. sobrinus*	*L. casei*	*A. naeslundii*
**CTRL_NEG**	2.0 × 10^5^	2.3 × 10^5^	1.2 × 10^5^	1.3 × 10^5^
**CTRL_POS**	<10	<10	<10	<10
**O_3__GAS80″**	<10	1.8 × 10^3^	4.6 × 10^3^	3.6 × 10^3^
**O_3__H_2_O80″ Simply**	3.1 × 10^3^	3.3	3.5 × 10^4^	<10
**O_3__H_2_O80″ Philozon**	2.2 × 10^3^	<10	<10	< 0
**O_3__H_2_O+GAS80″ Simply**	1.3 × 10^2^	<10	<10	3.3 × 10^3^

**Table 3:** Summary of the fracture analysis, showing the types of fracture found in each experimental group (in %).

Failure	Adhesive	Cohesive Dentin	Cohesive Resin	Mixed	Pre-Test
CTR	30.8%	11.5%	15.3%	15.3%	26.9%
O_3__GAS	30.9%	2.9%	27.9%	17.7%	20.6%
O_3__H_2_O	32.6%	2.2%	15.2%	17.4%	32.6%
O_3__H_2_O+GAS	28.8%	5.8%	17.3%	19.3%	28.8%

## Data Availability

The datasets presented in this article are not readily available because the data are part of an ongoing study. Requests to access the datasets should be directed to jcruz@egasmoniz.edu.pt.
